# Facilitators and Barriers to Newborn Sickle Cell Screening in Kalangala, Uganda

**DOI:** 10.5334/aogh.5382

**Published:** 2026-11-06

**Authors:** Madison K. Kitts, Joan Namata Perroni, Carol Nambwere, Julius Watema, Brian Herbert Ssemanda, Charmaine D. M. Royal, Stephanie O. Ibemere, Deogratias Munube, Joel Kibonwabake, Kearsley A. Stewart

**Affiliations:** 1 Duke Global Health Institute, Philadelphia College of Osteopathic Medicine South Georgia, Moultrie, Georgia, USA; 2 Kalangala Health Center IV, Kalangala, Uganda; 3 African & African American Studies, Biology, Family Medicine and Community Health and Duke Global Health Institute, Duke University, Durham, North Carolina, USA; 4 Duke University School of Nursing, Durham, North Carolina, USA; 5 Makerere University, Mulago National Referral Hospital, Kampala, Uganda; 6 Duke Global Health Institute, Duke University, Durham, North Carolina, USA

**Keywords:** sickle cell disease, newborn screening, Uganda, mixed-methods, barriers, facilitators, community perceptions, Kalangala District

## Abstract

*Background:* Sickle cell disease (SCD) is the most common inherited blood disorder worldwide. In Uganda, fifth in global prevalence, about 40,000 children are born with SCD annually. Newborn screening (NBS) enables early diagnosis and reduces mortality. Despite Uganda adopting universal NBS policies, little is known about factors affecting implementation in isolated areas. This study examined barriers and facilitators to NBS for SCD in Kalangala District, an island district in Lake Victoria, Uganda.

*Objectives:* This study investigates challenges and facilitators to Uganda Ministry of Health’s (MoH’s) call for universal NBS in a low-resource area and describes parent perspectives of NBS.

*Methods:* We conducted a mixed-methods study with surveys (N = 271) and in-depth interviews (N = 32) with parents of children under two years old about Kalanglala Health Center IV’s NBS program and their attitudes toward it. Data from surveys, in-depth interviews, and KHCIV’s annual NBS data assessed NBS capacity. Statistical analysis was conducted in RStudio and Excel; qualitative analysis was conducted in NVivo.

*Findings:* Among 271 participants, 83.8% had heard of SCD, but only 51.5% knew it is an inherited blood disorder. Most (61.6%) were unaware of the NBS at Kalangala Health Center IV, yet 81.5% would recommend screening. Most (51.3%) saw the first two days after birth as the best screening time. Of 455 records, SCD prevalence was 2.4%, higher than the national 0.7%. Qualitative findings highlighted limited knowledge, stigma, transportation, cost, and mistrust as barriers, while facilitators included trust in healthcare workers, positive screening experiences, and integration into community outreach.

*Conclusions:* Universal NBS for SCD is feasible in remote areas like Kalangala District, but barriers such as educational, geographic, financial, and health system issues threaten sustainability. Integrating SCD education into antenatal and immunization services, initiating community outreaches, and improving access to specialized care are key to nationwide scale-up.

## Introduction

Sickle cell disease (SCD) is a major global health burden, with over 75% of global cases occurring in Central, West, and East Africa [1]. Homozygous inheritance of sickle hemoglobin (HbS) causes sickle cell anemia (HbSS), one of the genotypes considered the most severe form of SCD [1]. Individuals with heterozygous inheritance (HbAS), referred to as sickle cell trait (SCT), may live their entire lives unaware that they have SCT [2].

Despite the widespread prevalence of SCD, obtaining reliable mortality and prevalence data is challenging [1]. Limited access to screening and care causes high mortality rates among children under five years of age across low- and middle-income countries (50–90%) compared with 5–10% in high-income countries [3–5]. Since 2000, global SCD births increased by 13.7%, with about 400,000 affected newborns annually, and are expected to rise further by 2050 [1, 4]. Reducing under-five mortality through early diagnosis and newborn screening (NBS) in Africa is crucial for lowering death rates. Infant mortality, especially before one year, is common in low- and middle-income countries, emphasizing the need for screening, as many infants are asymptomatic until their health declines [6]. SCD is a complex health issue influenced by structural and biological factors [7]. NBS is vital for understanding SCD in Uganda and for connecting diagnosed individuals to care, including therapy with hydroxyurea.

### Newborn screening in Africa

NBS is necessary for early detection of inherited and congenital disorders, ideally within 24–48 hours after birth [8]. NBS programs cover only about 33% of the global newborn population, yet higher-income countries screen for over 50 conditions [9, 10]. In contrast, congenital hypothyroidism is typically the only condition screened for in low- and middle-income countries [10]. The lack of universal SCD NBS across Africa has occurred due to prioritization of infectious diseases like HIV/AIDS, malaria, and tuberculosis, over non-communicable diseases (NCDs) like SCD [8]. However, NCDs are predicted to become the leading cause of death across Africa by 2030 [11].

Given generally high mortality rates and a growing population, concern about SCD as a public health threat prompted the WHO and the UN in 2006 to declare SCD a global health issue [10, 12]. That same year, the World Health Assembly recommended that African countries start NBS for birth defects and congenital conditions [11]. The WHO Regional Committee for Africa also adopted the NBS recommendation in 2006, leading to a strategy to tackle SCD [10]. In 2017, the American Society of Hematology started the Consortium on Newborn Screening in Africa (CONSA) to integrate NBS in Africa [8]. By 2020, CONSA had launched NBS programs in seven countries: Uganda, Ghana, Kenya, Liberia, Nigeria, Tanzania, and Zambia [8].

Uganda was among the first African countries to document SCD burden through the 2014 Uganda Sickle Surveillance Study (US3), which screened dried blood spots from HIV-exposed infants across 10 regions and 112 districts [1]. US3 mapped SCD trait prevalence at 13.3%, with eight regions showing over 20% SCT prevalence, shown in Figure 1 [1]. Population prevalence rates for SCD and SCT remain uncertain; however, prior studies focused on HIV-exposed infants, providing valuable data but leaving the broader newborn population unassessed [1].

**Figure 1:**
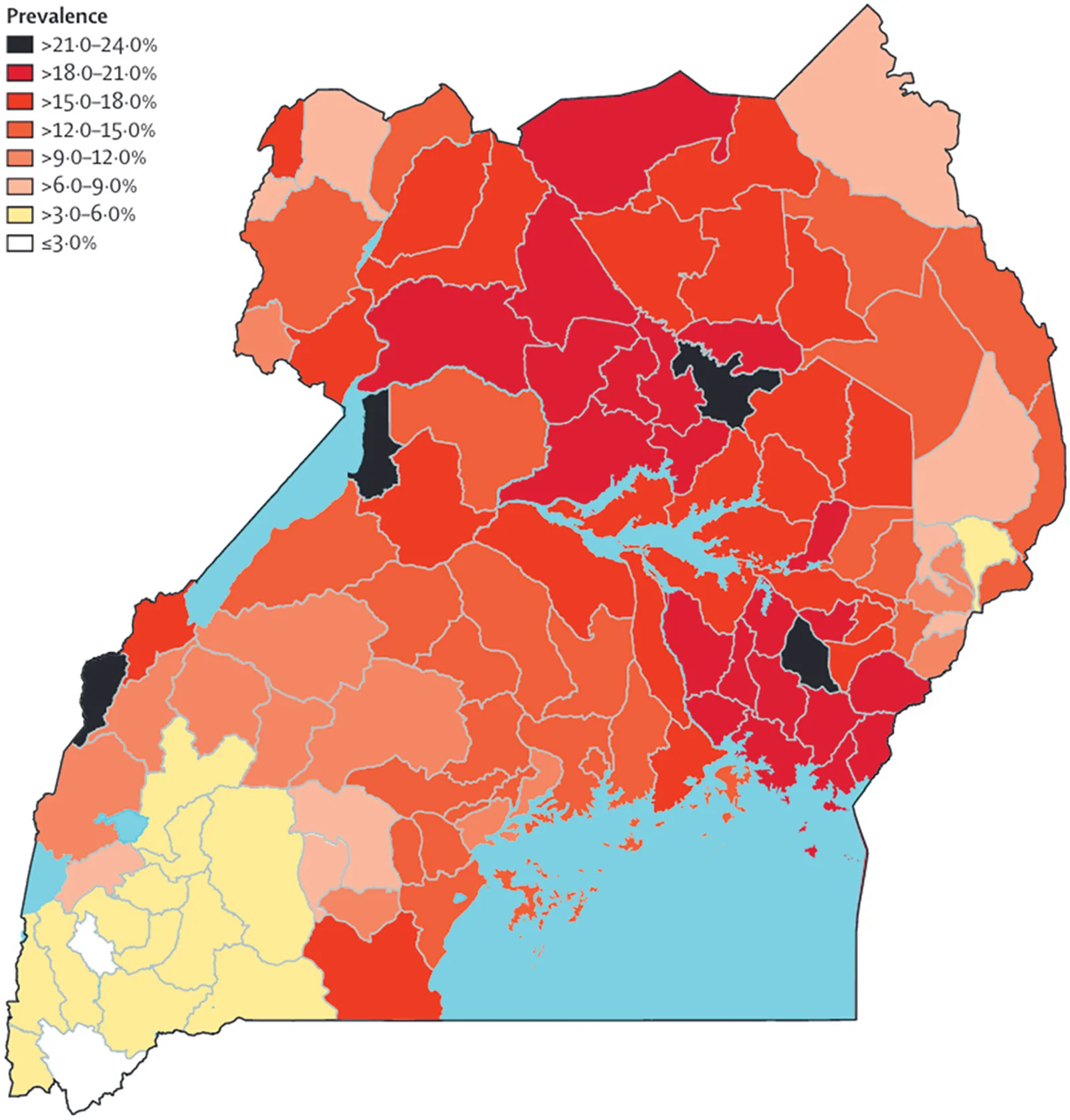
Map of sickle cell trait prevalence in Uganda [1]. This map shows the prevalence of SCT across 112 districts in Uganda from 2014 to 2015 [1]. SCT prevalence ranged from less than 3.0% to 24.0% [1].

NBS in Uganda was introduced by the Ministry of Health (MoH) following a National Sickle Cell survey in 2015, and as of February 9th, 2026, the MoH mandated NBS for SCD nationwide [13]. This policy provides free SCD screening at public health centers, and NBS protocols are to be implemented in 300 health centers [13]. The adoption of universal NBS in Uganda has been challenging, with slow uptake, limited infrastructure improvements, and insufficient resource support leaving health centers struggling to implement the policy [14]. However, partnerships with CONSA are ongoing within the country, and since 2021, the CONSA partnership has led to the screening of 29,080 infants and the confirmation of 419 SCD cases [15].

With 84.4% of the country’s population living in rural areas, many individuals choose to deliver with traditional birth attendants, as health centers or hospitals with the means to handle delivery and NBS can be hours of travel [13]. One such rural area is Kalangala District in the Ssese Islands. To better support NBS efforts in the Kalangala District, we conducted a mixed-methods study to characterize the current screening landscape at Kalangala Health Center IV through perspectives of parents whose children had been screened. Prevalence data for Kalangala District are difficult to determine as the district is aggregated into the Central Region [1]. Disaggregating Kalangala District from the Central Region is important for health surveillance, as the island community historically has among the highest prevalence rates for HIV and SCD [16].

## Methods

### Study design

From July 2024 to January 2025, we conducted a mixed-methods study using clinic data, parent surveys, and in-depth interviews with parents. Figure 2 shows both modules, from data collection to analysis. Clinic data included NBS at Kalangala Health Center IV, infant NBS results (N = 271), and the prevalence of SCD and SCT among all newborns screened (N = 455). The survey module (N = 271) examined knowledge of SCD and NBS, parental perspectives, and experiences. A subset of parents (N = 32) was recruited for interviews to discuss NBS experiences and attitudes toward SCD in communities and families. Participants were adult community members whose newborns or children were tested for SCD at the KHCIV Maternity Ward, Out-Patient Department, or laboratory after referral. An additional healthcare worker (HCW) interview module was conducted but is not included here; those results are in Kitts [17].

**Figure 2:**
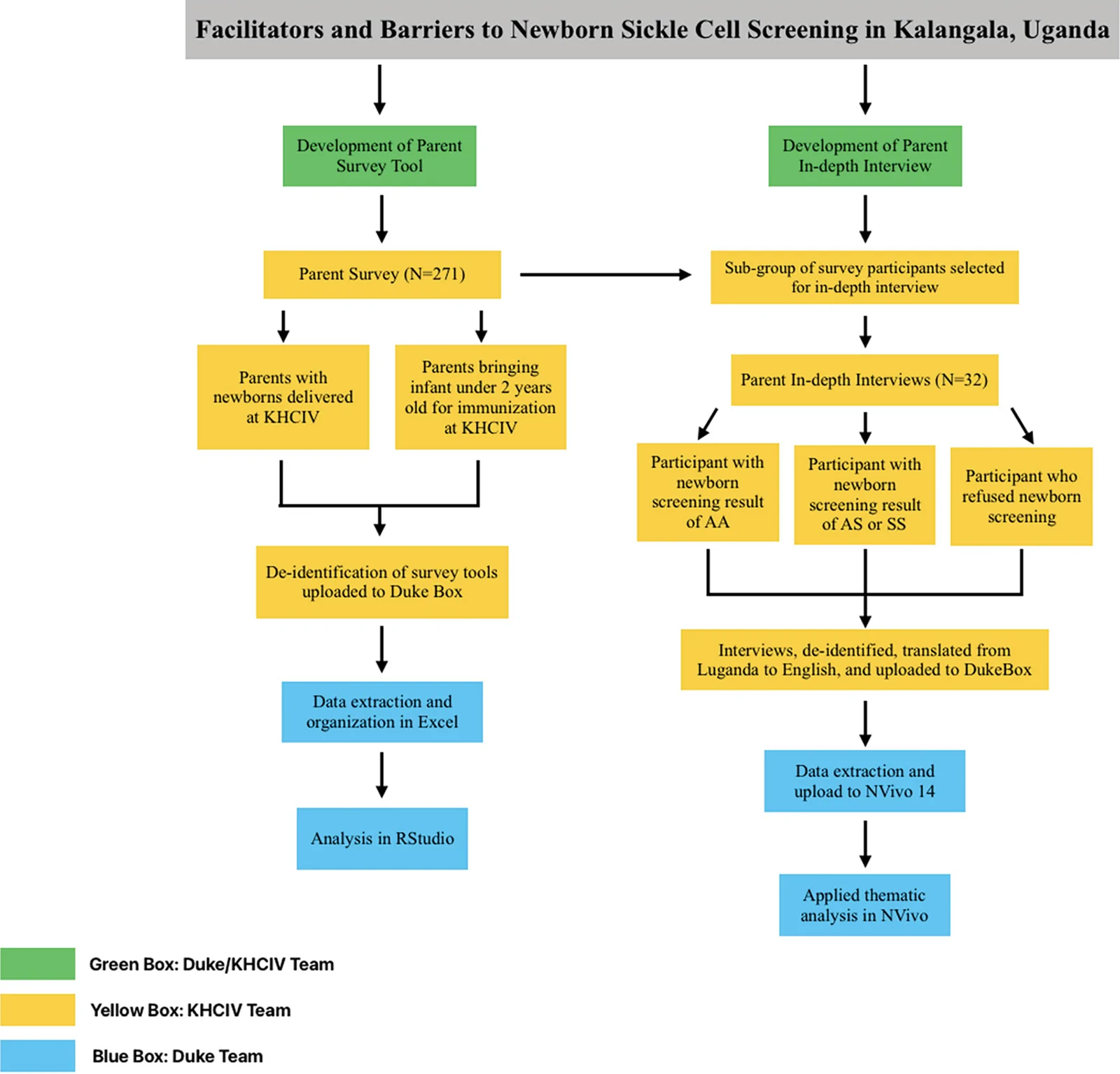
Study overview. A flowchart representing the process of our research from development of tools to final analysis.

### Kalangala Health Center IV (KHCIV)

This study was conducted at KHCIV, Uganda’s largest health facility in Kalangala District, an archipelago of 84 islands in Lake Victoria known as the Ssese Islands, with a population of about 74,411 across 63 inhabited islands [18, 19]. KHCIV is the main referral center for all 63 islands, but only 9 islands have government health facilities [18]. Due to its geographical isolation, KHCIV is designated by Uganda MoH as “Hard to Reach” because of infrastructural barriers and “Hard to Stay” due to poor living conditions and lack of amenities.

KHCIV offers outpatient, preventive, dental, obstetric, emergency, laboratory, and minor surgical services. In Uganda, sickle cell care is a specialized service available only at Level V hospitals. Specialized SCD services at KHCIV were unavailable until 2015, when clinician Joel Kibonwabake established the district’s first sickle cell clinic, which currently serves 129 patients from Buggala Island and the archipelago illustrated in Figure 3. KHCIV’s Sickle Cell Clinic team specializes in medication distribution, management, and counseling for caregivers of children with SCD and people living with SCD. KHCIV’s Maternity Ward averages 60 deliveries per month, and in February 2024, KHCIV was among the first Ugandan facilities to implement the MoH’s NBS.

**Figure 3:**
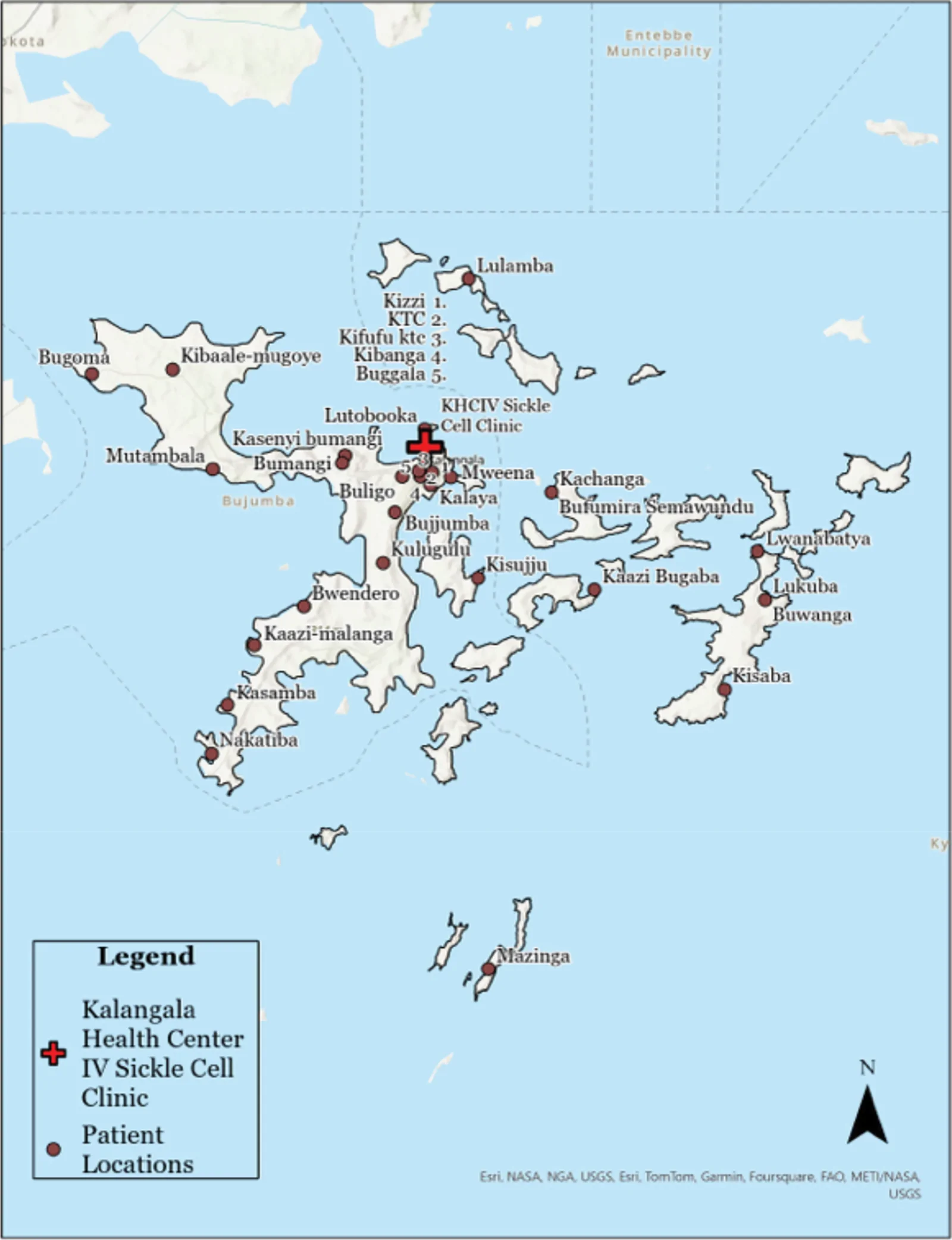
Map of sickle cell clinic and patient locations in Kalangala District. Patients’ residences in reference to the location of the KHCIV Sickle Cell Clinic (Map Credit: Jane Smoltz, 2024).

### Participant recruitment and data collection

#### Parent survey module

Data collection occurred from July 2024 to January 2025. All surveys were completed by January 2025, and a subset of NBS results that were previously unavailable was later located and incorporated. Participants, recruited by midwives and nurses at KHCIV, were parents of children under two years of age. Midwives approached new mothers post-delivery, and nurses in immunization clinics asked if their child had been screened for SCD (Figure 4). Parents unaware of screening were offered it and invited to join the study. Survey participants were recruited soon after testing, whether in the Maternity Ward or immunization clinics. The survey materials were the same for all.

Surveys were developed by Duke and Ugandan researchers at KHCIV Sickle Cell Clinic, including multiple-answer questions. Participants chosen for eligibility provided informed consent, received study numbers, and completed surveys in their choice of English or Luganda. The researcher read questions aloud for those who couldn’t read or write. The forms took about 25 minutes to complete. Results, translated by a native Luganda speaker, were entered into a spreadsheet.

#### Parent in-depth interview module

Participants who had completed the survey and had received testing results for their child were recruited to take part in interviews by the researcher who administered the initial survey. Interview participants were selected to represent AA, AS, and SS genotypes. None of our study participants received an NBS result of SS, so no SS cases are represented. Interview data collection ran from July 2024 to January 2025, and participants were a subset of participants from the survey module. Interviews examined beliefs, perceptions, and feelings about their newborn’s SCD status. The conversations were recorded using an audio recorder, and the entire process lasted about 60 minutes. The interviews were developed by the Duke and Uganda teams and conducted in Luganda by Ugandan research associates. Transcripts were then translated to English by a native Luganda speaker.

**Figure 4:**
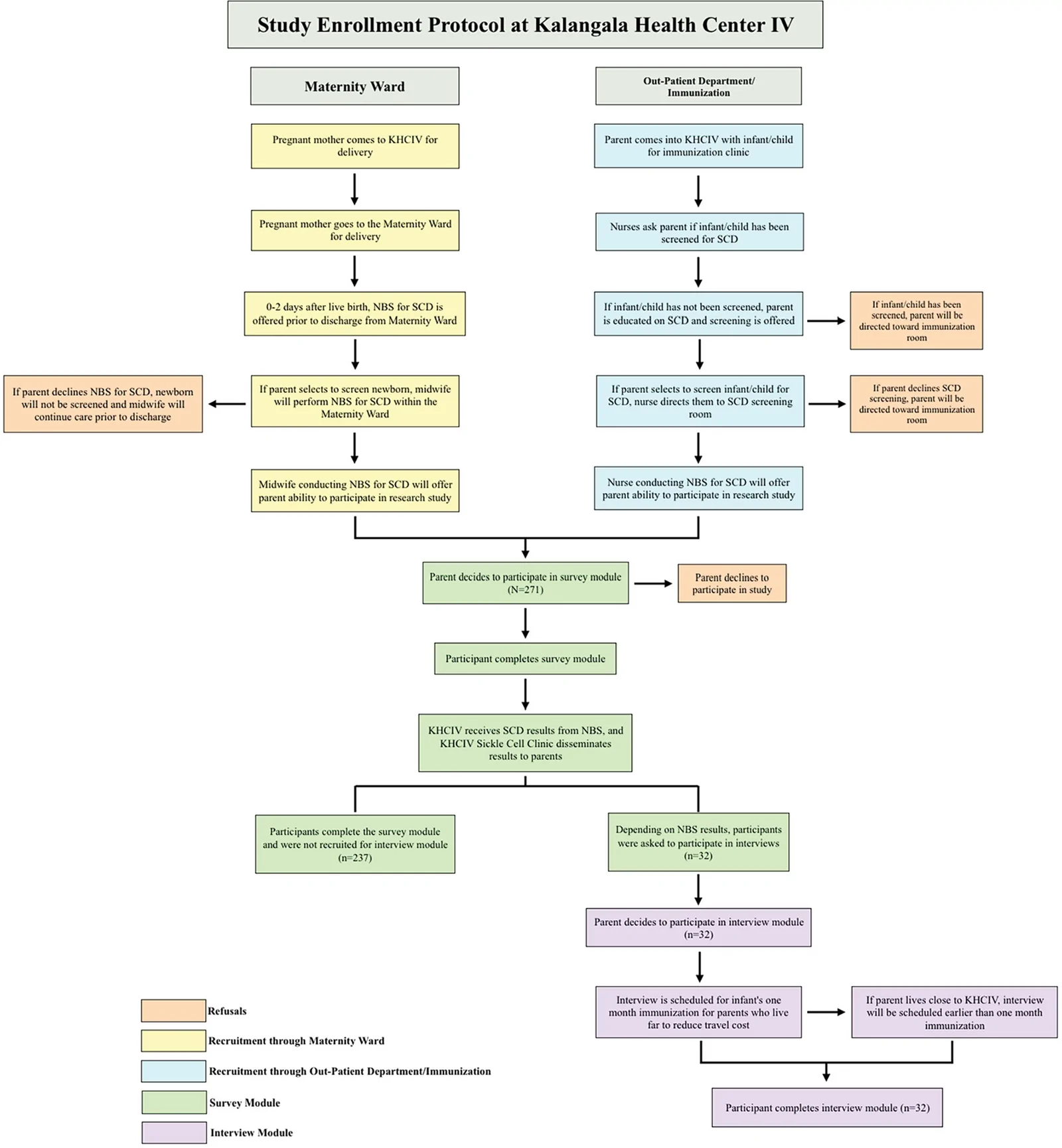
Study enrollment protocol at Kalangala Health Center IV. This flowchart demonstrates how individuals are recruited for both the survey and interview module. The two main pathways are through pregnant women delivering their newborns at KHCIV’s Maternity Department or parents coming to the Out-Patient Department for immunization clinic days.

### Data analysis

#### Statistical analysis

Survey data were entered into Excel and exported to RStudio (Version 4.3.1) for analysis. All survey results were complete, meaning all questions were answered, and we conducted non-parametric statistical analysis, including averages, percentages, and means on both survey data and demographic/medical data.

#### Qualitative analysis

The qualitative analysis identified barriers and facilitators to expanding newborn SCD screening through interviews with parents and HCWs (reported in Kitts [17]). All interviews were audio-recorded, transcribed verbatim, and translated from Luganda to English by a researcher from the local team. These transcripts were converted into Word documents, de-identified, and imported into NVivo (Version 14) for analysis. Using summative content analysis, key themes were identified through initial reading, coding, and grouping into broader themes [20, 21]. Data tables compared themes across participant groups, and quotes illustrated key findings.

## Results

### Parent surveys

A total of 271 participants (48 males and 223 females) completed the survey module. Demographic data are reported in Table 1. The birthplaces of our participants are shown in Figure 5, with Kalangala District having the highest number of participants (n = 36), followed by Masaka (n = 30).

**Table 1:** Demographic data of parents surveyed (N = 271).

	FEMALE (N = 223)	MALE (N = 48)
Age		
Median age in years	26	28
Age range in years	18–41	19–53
Education		
No Education	12 (5.4%)	1 (2.08%)
Primary school	74 (33.2%)	9 (18.8%)
Lower secondary (S1–S4)	107 (48.0%)	24 (50%)
Upper secondary (S5–S6)	11 (4.9%)	7 (14.6%)
Higher education/vocation	15 (6.7%)	6 (12.5%)
Other	4 (1.8%)	1 (2.08%)
Number of children		
First-time parent	62 (27.8%)	18 (37.5%)
Two children	79 (35.4%)	12 (25.0%)
Three children	50 (22.4%)	10 (20.8%)
Four children	24 (10.8%)	5 (10.4%)
Five or more children	8 (3.6%)	3 (6.3)
Birthplace by Region		
Central	112 (50.2%)	31 (64.6%)
Kalangala*	31 (13.9%)	5 (10.4%)
Northern	8 (3.6%)	3 (6.3%)
Eastern	25 (11.2%)	4 (8.3%)
Western	44 (19.7%)	5 (10.4%)
Other**	3 (1.3%)	0 (0.0%)

*Kalangala is not included in the Central Region total to represent the number of participants from the study location.

**Includes birthplaces outside of the country: Rwanda and the Democratic Republic of the Congo.

**Figure 5:**
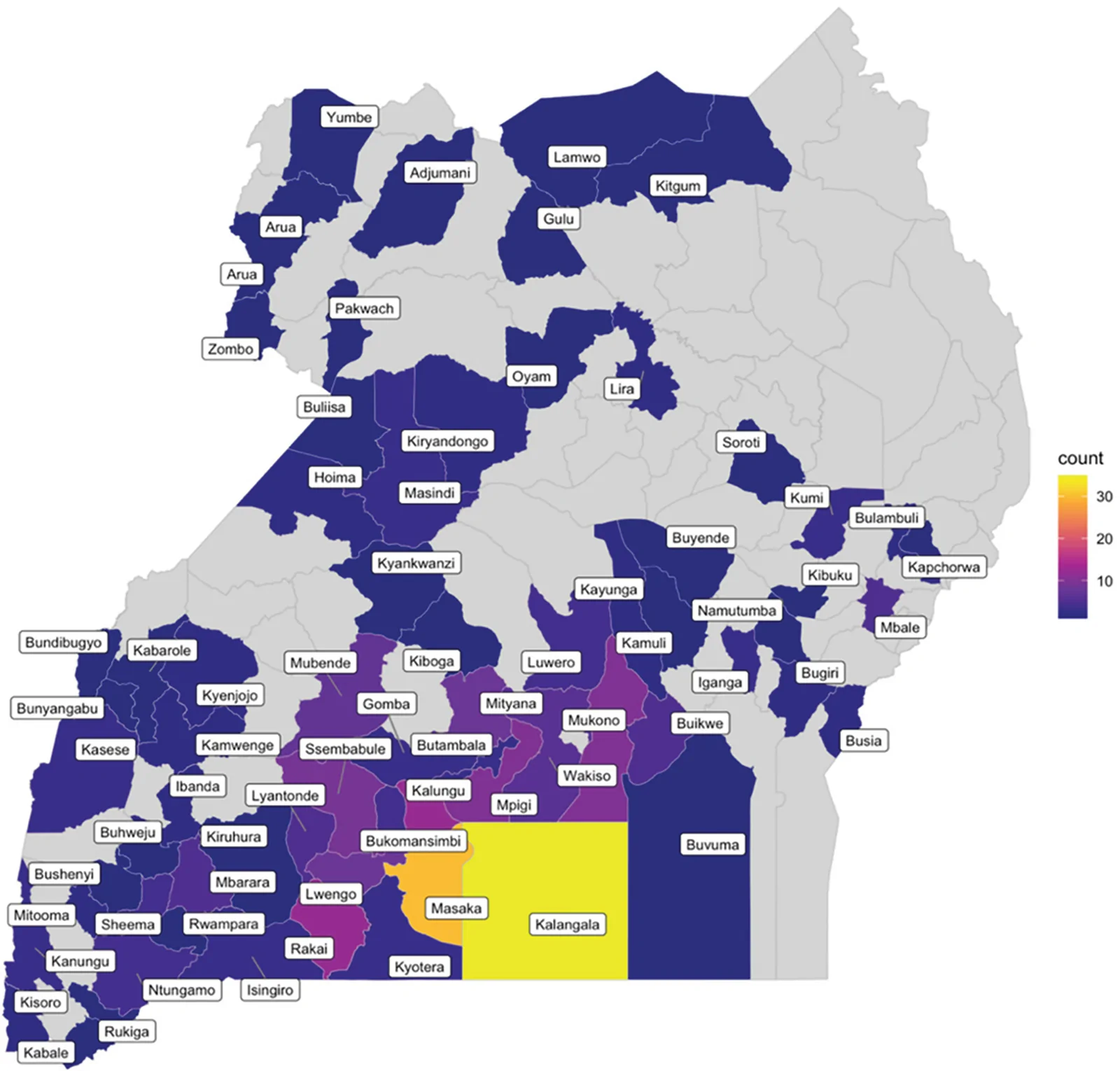
Map of participants’ birthplace in Ugandan districts. The map indicates the distribution of participants’ birthplaces throughout Uganda. Grey districts represent no data, while the color intensity of districts with data reflects the total number of participants.

### Newborn screening results for KHCIV and survey/in-depth interview participants

From February 2024 to January 2025, KHCIV’s Maternity Ward recorded 690 live births, of which 257 newborns were screened, yielding 37.2% NBS coverage within the Maternity Ward. Because NBS is available outside the Maternity Ward, we examined NBS testing across KHCIV’s three main screening departments: Maternity Ward, Out-Patient Department, and the laboratory. This analysis of NBS for all newborns and children under the age of two identified 455 screened at KHCIV. Most screens occurred in the Maternity Ward (n = 257/455, 56.5%), followed by the Out-Patient Department (n = 193/455, 42.4%). The remaining 1.1% (n = 5/455) were in the laboratory. SCD results, as shown in Table 2, indicate a prevalence of 2.4%.

**Table 2:** Newborn screening for SCD results for infants under two years old at KHCIV, February 2024–January 2025.

SCD STATUS	FREQUENCY	PERCENTAGES
AA	397	87.3%
AS	47	10.3%
SS	11	2.4%
Total	455	100.0%

Of the total number of NBS conducted at KHCIV (n = 455), participants in the survey and interview modules accounted for 59.6% of newborns and infants screened (n = 271/455) from July 2024 to January 2025. Among our participants, 8.1% (n = 22/271) of their newborns had the AS genotype, and the majority of those with the AS genotype were born to parents from the Central Region. None of the study participants’ newborns received an SS result. NBS results at KHCIV are summarized in Table 3.

**Table 3:** Participant newborn screening for SCD results for study participants, July 2024–January 2025.

SCD STATUS	FREQUENCY	PERCENTAGES
AA	249	91.9%
AS	22	8.1%
SS	0	0.0%
Total	271	100%

### Parent survey results

We analyzed 271 surveys. Participants were asked about their general knowledge of SCD, including awareness, inheritance, and treatment. Of the 271 participants, 83.8% (n = 227/271) reported having heard of SCD before the survey. Of the 227 participants who had heard of SCD, 51.5% (n = 117/227) accurately identified SCD on the survey as an inherited abnormality of the blood cells. Similarly, 49.4% (n = 134/271) of participants correctly identified the mode of transmission for SCD. Most participants (n = 167/271, 61.6%) were unaware of the ongoing MoH’s NBS program at KHCIV.

Participants were asked when a newborn should be screened, and 51.3% (n = 139/271) of responses indicated zero-to-two days after birth, aligning with current guidelines for NBS [22]. Additionally, 26.2% (n = 71/271) of participants thought newborns should be screened at the one-month check-up, and 10.7% (n = 29/271) responded that screening for SCD should occur at a six-month check-up.

Despite gaps in KHCIV’s NBS awareness campaign, survey results revealed that 81.5% (n = 220/271) were likely to recommend NBS for SCD. Regarding treatment, 77.9% (n = 211/271) would seek treatment at KHCIV if their newborn had SCD, while 11.4% (n = 31/271) would go elsewhere, excluding KHCIV, private facilities, or herbalists.

### Parent in-depth interviews

Participants in the interview module (N = 32) were selected based on their survey participation. Table 4 summarizes the demographic data collected from the sample participants.

**Table 4:** Demographic data of parents interviewed.

CHARACTERISTICS	FEMALE (N = 31)	MALE (N = 1)
Age		
Median age	25.13 years	27 years
Age range	18–35 years	27 years
Education		
No-literacy	4 (12.9%)	0
Primary school	8 (25.8%)	0
Lower secondary	13 (41.9%)	1 (100.0%)
Upper secondary & Higher education	6 (19.3%)	0
Number of children		
First-time parent	7 (22.6%)	0
Two children	15 (48.4%)	1 (100.0%)
Three children	8 (25.8%)	0
Four children	1 (3.2%)	0
Birthplace*		
Central Region	15 (48.4%)	0
Kalangala	2 (6.45%)	0
Eastern Region	4 (12.9%)	0
Northern Region	2 (6.45%)	0
Western Region	8 (25.8%)	1 (100.0%)

*Kalangala is not included in the Central Region total to represent the number of participants from the study location.

Themes in parent interviews were separated into two major categories: barriers (Table 5) and facilitators (Table 6). Themes were identified based on the frequency of the codes within the interview data. The results include a summary of the number of codes per theme, participant quotes, and demographic data.

**Table 5:** Barriers identified in parent in-depth interviews.

THEMES*	INTERVIEWS (N = 32)
Fear of Screening Process	15 (46.9%)
Fear of NBS procedures	10
Waiting for results	6
Little SCD Knowledge	28 (87.5%)
Fear of sickle cell disease	22
Lack of SCD education	23
Parental Identity and Family Instability	12 (37.5%)
Belief of AA status in partner	1
Domestic issues	11
Lack of Trust in the Health System	15 (46.9%)
Mistrust with blood samples	1
Belief KHCIV places little importance on NBS	2
Lack of HCW transparency	9
Distrust in HCWs	4
Physical and Financial Barriers	22 (68.8%)
Financial constraints	11
Inaccessible healthcare	12
Use of Traditional Birth Attendant(s)	4
Stigmatization and Isolation in Community	25 (78.1%)
Negative community perceptions	14
Stigmatization	23

*Number of interviews in sub-themes may not add up to the main theme total due to individual interviews containing multiple sub-themes, making one interview present across multiple data points.

**Table 6:** Facilitators identified in parent in-depth interviews.

THEMES*	INTERVIEWS (N = 32)
Ability to Access Healthcare	21 (65.6%)
Accessible healthcare	15
Screening during immunization	9
Prior Knowledge of SCD	22 (68.8%)
Parent aware of status	7
Prior experience with SCD	14
Received information on SCD	7
NBS Outreach and System Strengthening	31 (96.9%)
Using existing infrastructure	17
Ideas on improving NBS	29
Patient Confidence in Health Centers	32 (100%)
Trust in healthcare workers	17
Supports Ministry of Health (MoH) call for NBS	31
Positive experience with NBS	28
Community and Familial Support	17 (53.1%)
Support from family	6
Supportive behavior in community	15
Health-Seeking Behavior	31 (96.9%)
Engaging with midwives	10
Desire for more SCD knowledge	9
Wanting to know health status	29

*Sub-themes may not add to the main theme total due to individual interviews containing multiple sub-themes, making one interview present across multiple data points.

### The newborn screening process and trust in the health system

Fear of the NBS, from sample collection to getting results, was a major barrier for 46.9% (n = 15/32) of participants. Approximately one-third of participants (n = 10/32) said parents fear seeing their newborn in pain and see blood sampling as avoidable pain. This discomfort not only discourages NBS but also routine immunizations.

“*They fear to hurt their babies and also hate the discomfort the babies have to go through after the test*.” (Female, 28 years old)

However, 87.5% (n = 28/32) of participants reported a positive experience with NBS, despite the fear and hesitation that accompany the process. It is important to acknowledge apparent contradictions to demonstrate the complexities of awareness and protocol implementation. While parents may not want their newborn or infant to be in pain, many were surprised that their newborn was not in discomfort for long, making the experience more positive than previously believed.

*“The process wasn’t bad. The baby didn’t seem hurt, and they were really quick.”* (Female, 23 years old)

The waiting period for results impacted attitudes toward NBS. Six of 32 participants noted that the long wait was especially problematic for those on remote islands. Results take from one week to a month after sample collection and are sent to KHCIV. Clinicians or lab staff contact parents afterward, but issues like poor cellular coverage, limited electricity, and transportation challenges are common.

*“It is long and a little inconveniencing for people who live in remote islands. They would struggle to get their results since they rarely come to this side.”* (Female, 20 years old)

Many participants (n = 15/32) expressed fear of the screening process, linked to mistrust in health centers and HCWs. Mistrust arose from perceptions of blood sample misuse (n = 1/32), lack of transparency (n = 9/32), beliefs that KHCIV undervalues NBS (n = 2/32), and overall distrust in HCWs (n = 4/32).

*“People know each other, and they are scared of rude health workers.”* (Female, 20 years old)

*“[The healthcare worker] didn’t brief me on how the sample was to be collected. I didn’t know they were going to prick the toe. I was a little scared.”* (Female, 34 years old)

Conversely, 53.1% (n = 17/32) of our participants indicated trust in HCWs, and an overall belief that those working at KHCIV are a reliable source of information and care. Both positive and negative experiences with HCWs are essential to document, as these experiences directly impact the way a patient views healthcare and their health-seeking behaviors.

*“I only think they should teach us more about sickle cell disease before testing. They asked parents if they wanted to test their children for SCD. How do I test for something I don’t know, or I can’t explain if go back to my husband.”* (Female, 20 years old)

*“I think it accords you a certain level of privacy. Unlike in the community, where people can be nosy, here [KHCIV], you have some confidentiality.”* (Female, 34 years old)

### SCD Knowledge and health-seeking behaviors

Most participants (n = 28/32) identified limited SCD knowledge as a barrier to NBS, citing fear of the disease (n = 22/32) or lack of SCD education (n = 23/32). Surveys show 83.4% have never been tested for SCD, and 90.2% are unaware of their partner’s status. However, participants highlighted the difficulty of persuading partners to get tested [23].

*“Most men don’t bear the responsibility of children, and they are very hesitant to participate in such programs.”* (Female, 24 years old)

Some participants (21.8%, n = 7/32) said stigmatization of SCD testing leads people to avoid testing to prevent HIV suspicion or isolation. The community links SCD and HIV/AIDS, using the same screening tools, fueling misinformation about SCD [23].

*“Just like people fear to test for HIV because they are scared to take lifelong medication and also to deal with the shame that comes with it.”* (Female, 18 years old)

In aware families, individuals trusted NBS and saw its importance. 21.8% (n = 7/32) of those knowing their SCD status were motivated to test their newborns. Community campaigns with free testing and education could dispel misconceptions and encourage testing. Additionally, 43.8% (n = 14/32) had prior SCD knowledge, boosting their willingness to participate in NBS.

*“Men don’t spend time with these kids, it is us women who are always with them. We take heed in everything! Even if it is HIV, the woman will be the one to make sure the child doesn’t get infected”*. (Female, 35 years old)

### Community and familial perceptions and stigmatization

Stigmatization of SCD is a major barrier to NBS and community health, significantly affecting parental screening decisions and care. 71.9% (n = 23/32) of participants stated their community and family would abandon them if they knew their child had SCD.

*“I can’t tell people in the community. They would mistreat her, and I don’t want to watch my child go through that.”* (Female, 28 years old)

Others expressed that their child would experience social isolation from their peers at school if their classmates knew their status. Among the most concerning, participants revealed that community members and peers will tell a person living with SCD that they will die early or at any time after diagnosis.

Despite 71.9% of participants reporting experiences of stigma, over half (53.1%, n = 17/32) believe proper education would prevent stigma for their child. Supportive community and family members are those aware of SCD and with prior education. Of the 17 willing to disclose their child’s SCD, 6 would only inform family, mainly in health emergencies.

*“In case of an emergency, they [my family] would do the right thing, but if you don’t tell them, everyone will administer any drug to the child if you are absent.”* (Female, 28 years old)

Complexities among intimate partners affected 34.4% (n = 11/32) of interviews. In Kalangala, women delayed seeking health due to domestic and sexual violence [24]. Men view health spaces as female spaces, limiting their involvement in antenatal care and NBS. Domestic roles and fear of blame hinder women from asking partners about SCD [25]. Partners and husbands often blame the mother’s lineage, placing testing and care burden on women. Many show disinterest in screening for SCD or their children.

*“I have been so worried all this time waiting for [the results] because I am a carrier, and I don’t know my husband’s status. I wish I could get someone to convince him to get tested because I feel like we risk with every birth. Once I get to know his status, I can decide to stop giving birth. He only cared about HIV, but I hope one day he tests.”* (Female, 29 years old)

*“My husband wasn’t happy I tested my child for sickle cell disease. He said, “Congratulations on looking for new trouble.” He said good thing he doesn’t stay with us and, and if the results are positive, I would face it.”* (Female, 28 years old)

### Community health outreaches and education

Challenges related to cost and access to a health facility with NBS were prominent. Financial constraints (n = 11/32) limited individuals’ access to healthcare, as travel costs to KHCIV vary widely by island of origin. At the KHCIV Sickle Cell Clinic, 30.3% of patients travel an average of 4 or more hours to the clinic (per unpublished data collected by Krysten Rose-McCully). Additionally, 22% of patients spend between 10% and 50% of their weekly income on travel, and 43% spend more than 50% (per unpublished data collected by Krysten Rose-McCully).

*“There are so many factors that bar people from coming to health centers, for instance, remoteness and high transport fares.”* (Female, 29 years old)

High travel costs are just one of the financial barriers addressed by participants, with the ability to access healthcare being another challenge for this island community. Remoteness, limited capacity to existing outreaches, and lack of available medicines were identified by 34.4% (n = 11/32) of participants, while 46.9% of participants reported they had access to NBS health services, particularly through immunization programs. Immunization day, a weekly program held on Thursdays at KHCIV, was identified as a key educational component of the current NBS services offered at KHCIV, motivating parents to screen their newborns or infants.

*“I have never heard anyone in my community talk about SCD testing. I actually first heard about SCD testing here when I came to immunise. I had never heard about it in my entire life.”* (Female, 20 years old)

While nine participants saw newborn immunization outreaches as a viable NBS option for remote communities, some (n = 3/32) felt these were their only testing opportunity, which they were unable to do.

*“There are people who give birth at traditional birth attendants and those who don’t come for immunisation. They all need to be reminded to take part in these programs.”* (Female, 20 years old)

## Discussion

This study on NBS for SCD in Kalangala, Uganda, found higher SCD prevalence at KHCIV, financial and geographic barriers, and a need for community education and stigma reduction. NBS results (Table 2) show a 2.4% SCD prevalence, three times the national rate of 0.7% [1]. This underscores the need for awareness campaigns that guide communities to make informed health decisions about SCD screening, treatment, or support [24]. Survey findings suggest that proactive community attitudes toward timely NBS, within 24–48 hours after birth, could reduce access and travel costs, allowing individuals to participate in screening.

Across all 271 participants, 51.5% correctly identified SCD as an inherited red blood cell disorder in a multiple-choice survey. Despite 83.3% of participants indicating prior awareness of SCD, survey results showed limited knowledge about SCD, consistent with Tusuubira et al. [26]. This study found that 91.2% of adults living in Kampala, Uganda, knew of SCD, but 44.2% did not know its cause [26]. By comparison, in Nigeria, 98.4% of participants knew of SCD, but only 17.8% had adequate knowledge, and in the United States, 79% of participants knew of SCD, while 21% had no prior knowledge [27, 28].

NBS differs from NBS awareness, which focuses on education and encourages screening for SCD. Interviews conducted early in the NBS rollout at KHCIV may explain participants’ prior lack of awareness of the NBS program. Most survey participants supported SCD education during antenatal care visits (78.2%, n = 212/271), suggesting expanding SCD education would meet a community demand. While the MoH has revised current guidelines, SCD screening is not integrated into Uganda’s antenatal care package across all districts, and education remains unstandardized [22, 23]. This represents a clear opportunity for policy expansion.

Stigma and gender roles remain barriers to screening. In previous studies of secondary school students in Kampala, 83.2% agreed partners should disclose their status before intercourse or marriage [23]. Women are often blamed for the disease, and burdened with child-rearing and health-seeking responsibilities, with screening for SCD being seen as “looking for trouble.” While most participants wanted SCD education during visits, 14% preferred information upon request, showing a conflict between education and patient autonomy. While stigma and gender roles are real barriers, religion is shown as a major coping mechanism for people living with SCD [29]. The partnership with local religious leaders to help distribute SCD information and promote premarital screening could be useful in combating stigma and gender-based blame.

Previous immunization outreach efforts were delayed or canceled due to supply shortages, transportation costs, or weather [25]. These immunization outreaches often represent the first contact with a skilled clinician for newborns, as many women opt for traditional birth attendants to avoid travel. Travel costs to KHCIV reflect the struggle families face in health access [30]. If an infant is detected to have SCD, parents will experience an increase in travel costs for medication and potential hospitalizations. Text messaging was found to be parents’ preferred method for receiving NBS results, though data costs, health staff workload, and parent interpretation remain challenges [31].

The study’s findings inform policy and practice for universal NBS for SCD. Surveys and interviews identified challenges, including educational gaps, stigmatization, and misinformation. With increased attention to SCD through health policy development and the mandating of screening programs in Uganda, decentralizing SCD services and community programs involving traditional birth attendants could promote NBS-seeking behaviors among parents [7, 30]. Similar awareness efforts in Uganda increased service uptake; thus, incorporating community workshops into SCD screening could enhance NBS participation [32]. The recent national NBS mandate raises concerns about limited access to hydroxyurea and the adequacy of the National Medical Store’s response as more infants are diagnosed. As NBS expands, clearer ethical guidance on costs and readiness is needed to ensure equitable access. Hernandez et al. [33] show that high initial costs for early diagnosis reduce long-term expenses. Incorporating SCD education into antenatal care could promote early detection and awareness, lowering costs associated with late diagnosis and complications [33, 34]. To respect patient choice, midwives might briefly introduce SCD and offer additional information if desired. Standardized educational materials and MoH training are essential for effective NBS education.

The MoH recently launched Africa’s first HU manufacturing plant to improve access to medications like hydroxyurea [35, 36]. Supporting local solutions boosts early treatment and supply chains, both crucial for NBS programs and building trust. Combining NBS with immunization improves access, but limited infrastructure at SCD clinics, especially when KHCIV is the sole provider, hampers this, impacting remote islands. Uganda needs MoH involvement, funding, and public–private partnerships for nationwide NBS. Caregiver education on SCD and early symptoms is essential, as some parents deny positive results, risking severe outcomes. Explaining hydroxyurea and treatment access at KHCIV shows early intervention reduces mortality. Engaging parents also enhances advocacy.

### Limitations

The sample was restricted to children aged two years and below at a single health center; therefore, our SCT and SCD prevalence rates might not represent the broader district population. Additionally, midwives and nurses recruited participants during clinic hours, and refusals were not systematically recorded for survey module participants.

## Conclusion

Our main goal was to gather perspectives on NBS to identify support areas in the health system and community. Surveys, interviews, and NBS results show that NBS at KHCIV is critical for health education and early diagnosis. Parents and HCWs at KHCIV, Mulabana Health Center II, and Bwendero Health Center III recommend immunization outreach to screen children not born at facilities offering NBS. Kalangala District faces healthcare and education delivery issues to nearby islands. Reports of samples being thrown overboard due to unstable boats highlight infrastructure needs to improve travel and transfer of medical services [24].

Given the island’s geography and high SCD burden, we recommend the MoH promote a safe work environment and transportation for outreaches by purchasing updated boats, ideally, boat ambulances, for health centers. Reliable transport encourages HCWs’ participation, and secure equipment like a Gazelle for rapid SCD testing would enable remote islands to access NBS programs [37].

With NBS now required at Ugandan health centers, parents can give feedback on policy and service quality. Our study calls for future research, including the cost-effectiveness of parental involvement in NBS in Kalangala District. Future studies should explore community health workers’ and traditional birth attendants’ roles in promoting NBS and SCD campaigns, and how health centers implement the MoH’s NBS mandate [37]. Additionally, studies could explore integrating community and spiritual support into SCD counseling by partnering with local religious leaders. Understanding gender biases and fathers’ involvement in NBS for SCD is another important area for future study.

Community education remains a key goal, focusing on families through counseling and campaigns using trusted networks [38]. Immunization and screening outreaches improve NBS access in this remote district, and such programs should be led by the MoH in close collaboration with clinicians and community health workers. Our findings highlight the need to consider community and parent perspectives on health programs. Their ongoing involvement is essential in health promotion and education.

## Figures and Tables

**Figure:**
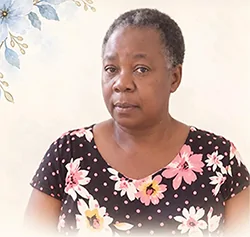


## Dedication

We dedicate this paper to the late Sister Annet Mutiibwa, the Senior Nursing Officer of Midwifery at Kalangala Health Center IV. Sister Annet dedicated her life to serving others, and we will feel her loss every day. May she continue to inspire and motivate us to advocate for healthcare in underserved areas, and may we do so with the joy and fearlessness she displayed.

## Additional Files

The additional files for this article can be found as follows:

Supplementary material 1. Parent_Interview_English.

Supplementary material 2. Parent_Survey_English.
