# Supplementary material for: Facilitators and Barriers to Newborn Sickle Cell Screening in Kalangala, Uganda
Source: Ann Glob Health. 2026 Nov 6;92(1):109. doi: 10.5334/aogh.5382 (PMC13741941; doi:10.5334/aogh.5382)
Supplement: Supplementary file 1 [file agh-92-1-5382-s1.pdf]

7.1.1.3 In-Depth Interview Questions for Parents - English

Demographic Data

1	Participant Number	
2	Gender	
3	Age	
4	Place of birth	
5	First language spoken	
6	What level of school did you complete?	
7	When did you leave school?	

1. If you did test your child for sickle cell disease, can you explain why you did?
    - *What was your experience with the testing process?*

2. What was most concerning about the testing process for you?

3. What was one aspect of the testing process that you thought was helpful?

4. Do you think that the hospital places an importance on sickle cell disease testing?

5. The Ugandan government is calling for all children to be tested for sickle cell disease. Do you believe this goal is a good idea?
    - *What are the possible benefits of this program?*

6. Was your decision to test for sickle cell disease influenced by personal experiences, and can you explain those experiences?

7. In your community, how are people with sickle cell disease treated?
    - *What are the common myths about sickle cell disease testing in your community?*

8. How best can we educate people about SCD newborn screening?
    - *What are the available models or channels we can use to quickly inform the public about newborn screening of SCD?*

9. What segment of people do you think should be targeted most during this campaign?

10. How best can parents be encouraged to adhere to this new program of NBS?

11. If there was one thing you would change in your community regarding sickle cell disease, what would it be?

12. If you had a child with sickle cell disease, how would your family and community treat them?

13. How best do you think we can scale up this program to benefit your community?

14. Is there anything I forgot to ask but I should know about you and your perspective on sickle cell disease testing?
