# Supplementary material for: Facilitators and Barriers to Newborn Sickle Cell Screening in Kalangala, Uganda
Source: Ann Glob Health. 2026 Nov 6;92(1):109. doi: 10.5334/aogh.5382 (PMC13741941; doi:10.5334/aogh.5382)
Supplement: Supplementary file 2 [file agh-92-1-5382-s2.pdf]

7.1.1.1 SCD Survey Questions for Parent(s) - English

Demographic Data on Parent

1	Participant Number	
2	Gender	
3	Age	
4	Place of birth	
5	First language spoken	
6	What level of school did you complete?	
7	When did you leave school?	

Demographic Data on Child

1	When was child born?	
2	Child's sex	
3	Child's weight	
4	Do you have any other children?	
5	If yes, note their age and sex.	

Checklist on Newborn Screening Testing

1	Were you offered a sickle cell test?	
2	Did you accept or deny the test?	
3	If you did not accept, explain why.	
4	If you did accept, do you have the results?	
5	Tracking number for sickle cell test	
6	Date of test	

*Participants can choose more than one answer

1. Have you ever heard of sickle cell disease?
   a. Yes
   b. No
2. What is sickle cell disease?
   a. A group of inherited red blood cell disorders

   b. Healthy round red blood cells
   c. Abnormal shape red blood cells
   d. I do not know
3. How does someone catch sickle cell?
   a. Touch
   b. Hereditary
   c. Bodily fluid
   d. I do not know
4. How is sickle cell disease diagnosed?
   a. Blood test
   b. Body scan
   c. Urinary test
   d. Temperature test
   e. I do not know
5. Which one prevents sickle cell disease?
   a. Medical advice
   b. Pre-marital screening
   c. Drugs
   d. I do not know
6. Is there a cure for sickle cell disease?
   a. Yes
   b. No
   c. I do not know
7. What is the treatment for people with sickle cell disease?
   a. Medicines
   b. Proper diet
   c. There is no treatment for sickle cell disease
8. Do you know anyone with sickle cell disease?
   a. Yes
   b. No
   c. I don’t know
9. In a situation where a child is born, should they be tested for sickle cell?
   a. Yes
   b. No
   c. Only if requested by the parents
   d. I don’t know
10. Do you know anyone with sickle cell disease?
    a. Yes
    b. No
    c. I don’t know.
11. If applicable, who do you know with sickle cell disease?
    a. Sibling
    b. Parent
    c. Both parents
    d. Uncle
    e. Aunt
    f. Niece
    g. Nephew
    h. Grandmother
    i. Grandfather
    j. Child
    k. Self
    l. Family member not listed

12. Did you know about SCD newborn screening at KHCIV?
    a. Yes
    b. No
13. If both parents have sickle cell disease can their children get sickle cell?
    a. Yes
    b. Maybe / unsure
    c. I do not know
14. If your newborn had SCD, where would you first seek treatment?
    a. At KHCIV
    b. Private health facility
    c. Traditional herbalist
    d. Other
15. A mother has just given birth to a newborn girl. When do you think the child should be screened for sickle cell disease?
    a. 0-2 days after birth
    b. At one month check up
    c. At 6 month check up
    d. At one year check up
    e. I don't know
16. A mother goes to the hospital for prenatal care. Do you think healthcare workers should provide information on sickle cell disease testing for her child?
    a. Yes
    b. No
    c. Only if it is requested
    d. I don't know
17. Are you likely to recommend a person you know to screen their infants for SCD?
    a. Very likely
    b. Likely
    c. Not likely
